# Bladder mass following bone marrow transplant for sickle cell disease: A diagnostic dilemma

**DOI:** 10.46989/001c.161296

**Published:** 2026-05-04

**Authors:** Yusuf Adelabu, Ugonna O. Fakile, Wale Q. Oladimeji, Kayode Oseni, Moses A. Ogunjimi, Titilayo G. Bamigboye, Blessing N. Aziken-John, Amina Enegbuma, Zainab Olayiwola, Olufunto Kalejaiye, Titilope A. Adeyemo, Ann Ogbenna, Karina Wilkerson, Edamisan Temiye, Adeseye M. Akinsete, Adetola A. Kassim

**Affiliations:** 1 Lagos University Teaching Hospital, Idi-Araba, Lagos, Nigeria; 2 Department of Medicine, Faculty of Clinical Sciences, College of Medicine, University of Lagos, Lagos, Nigeria; 3 Department of Paediatrics, Faculty of Clinical Sciences, College of Medicine, University of Lagos, Nigeria; 4 Department of Surgery, Faculty of Clinical Sciences, College of Medicine, University of Lagos, Lagos, Nigeria; 5 Sickle Cell Foundation Nigeria, Idi-Araba, Lagos, Nigeria; 6 Department of Haematology & Blood Transfusion, Faculty of Basic Clinical Sciences, College of Medicine, University of Lagos.; 7 Department of Medicine, Division of Hematology/Oncology, Vanderbilt-Meharry Center for Excellence in Sickle Cell Disease, Vanderbilt University Medical Center, Nashville, TN, USA

**Keywords:** Sickle cell disease, bone marrow transplantation, hematuria, bladder mass, inflammatory pseudotumor

## Abstract

Hematuria is a common sign of kidney and urinary tract issues in sickle cell disease (SCD). Hematopoietic stem cell transplantation (HSCT) can cure SCD but may also be complicated by conditions that cause hematuria. We report a case of painless hematuria caused by a bladder mass in a young woman after allogeneic HSCT for SCD. Further evaluation showed a friable pseudotumor of the bladder, which was deemed benign based on histological examination. The hematuria resolved with continuous bladder irrigation and cauterization of the bleeding mass. This case highlights that hematuria can originate from hidden pre-existing bladder lesions in the post-transplant setting.

## Introduction

Sickle cell disease (SCD) is an inherited hemoglobin disorder characterized by hemolysis and vaso-occlusion, leading to multiple acute and chronic complications.^1^ Allogeneic hematopoietic stem cell transplantation (HSCT) is a curative treatment for children and adults with severe SCD.^2^ Both SCD and HSCT can be associated with various renal and urological complications, including acute and chronic kidney injuries, infections, inflammatory conditions, and neoplastic issues.^3,4^ A common urologic complication in SCD is painless hematuria, which results from papillary necrosis caused by hypoxic tubular injury in the renal medulla.^5^ This symptom is usually alarming to patients, their families, and clinicians. Following HSCT, acute kidney injury typically occurs within the first 100 days, with an incidence rate of 21-84%, and the highest occurrence is observed with myeloablative conditioning.^6^ Hemorrhagic cystitis secondary to BK virus and adenovirus infections is a common cause of delayed hematuria after HSCT.^7^ It is crucial to thoroughly evaluate individuals with hematuria following HSCT and intervene appropriately, as most cases are self-limiting but can be life-threatening.^7^ In this article, we present a case of delayed-onset hematuria caused by a bladder mass in a young woman who underwent allogeneic bone marrow transplantation (alloBMT) for severe SCD.

## Case report

A 17-year-old female with SCD had an allogeneic bone marrow transplant (alloBMT) in June 2025 from a matched sibling donor. She received a myeloablative conditioning regimen including oral busulfan at a total dose of 14 mg/kg, administered every six hours for 16 doses from days -9 to -6, intravenous (IV) cyclophosphamide at 50 mg/kg/day on days -5 to -2, and antithymocyte globulin at 2.5 mg/kg/day on days -4 to -2. She was hyperhydrated with 3 L/m2/day of IV fluids and given Mesna 16.7 mg/kg at 0, 3, and 6 hours after cyclophosphamide on days -5 to -1 to prevent hemorrhagic cystitis. Both the donor and recipient were cytomegalovirus seropositive (IgG) at the time of transplant. The recipient tested positive for hepatitis B core antibody. Post-transplant, she achieved neutrophil engraftment on day +13 and platelet engraftment by day +15. However, her immediate post-transplant course was complicated by Klebsiella sepsis and Clostridium difficile infection treated with meropenem and metronidazole, acute graft-versus-host disease (GVHD) treated with oral budesonide, and acute medication-induced psychosis managed with risperidone, haloperidol, and benzhexol (Artane). She also maintained immunosuppression with oral tacrolimus at 1.5 mg daily, achieving good serum levels. Menstrual prophylaxis was established with oral medroxyprogesterone 3 mg daily, and prophylaxis against hepatitis B viral reactivation was provided with oral tenofovir 300 mg daily, along with other routine antimicrobial agents, while being routinely followed in the clinic three days a week post-engraftment.

She developed a sudden, painless hematuria on day +64 post-transplant, with passage of blood clots in her urine. There was no associated fever, dysuria, urgency, or urinary frequency. She had no lower abdominal or flank pain. She had no previous episodes of hematuria. The clinical examination at the time of hematuria did not reveal any significant findings. There was no suprapubic or renal angle tenderness, and no palpable abdominal organs or masses. Initial complete blood count following the appearance of hematuria showed a hemoglobin level of 11 g/dL, a platelet count of 50,000/μL, and a whole-blood RFLP of 80% donor. The clotting profile was essentially normal. Urinalysis confirmed the presence of blood (3+) without protein. Urine microscopy revealed intact red blood cells (>100 per high-power field), with no cell casts or parasite ova (**Table 1**). The urine culture showed no bacterial growth. Quantitative viral studies on urine for adenovirus and BK virus did not detect any viral particles. The renal ultrasound scan showed no abnormalities.

**Table 1.** Time course of gross hematuria and summary of the investigations and interventions

Day post-transplant	Investigation	Findings	Intervention
+65	Urine macroscopy and microscopy	Frank blood in urine with clots Numerous intact red blood cells (>100/high-power field) No bacteria, yeast cells, crystals, casts, or ova of parasites	CBC monitoring Transfusion support Continuous bladder irrigation
+65	Urine culture Viral screen (Adenovirus, BK virus, CMV, and EBV)	No bacterial growth No viral particles detected	Antibiotic prophylaxis
+65	Renal ultrasound scan	No abnormality detected	
+100	Cystoscopy	Hyper-vascularized bladder wall with two coral reef-like exophytic bladder masses in the right and left anterolateral wall, with bleeding points	Cauterization Bladder wash-out Biopsy of masses
+102	MRI of the bladder	Diffuse bladder wall thickening with a focal lesion	
+106	Histology of the biopsy from the masses	Dense stromal infiltration by inflammatory cells, mainly lymphocytes, suggestive of bladder pseudotumor	Conservative management (liberal fluid intake and observation)

She was admitted the day after experiencing hematuria (day +65), which involved the passage of large blood clots. She started hydration with normal saline at 62 mL/hr, later increasing to 83 mL/hr, then 100 mL/hr, and finally 167 mL/hr. She also received transfusions of irradiated red cell concentrates after her hemoglobin dropped to 8 g/dL. Following a urology review, a three-way silicone hematuric catheter was inserted for continuous bladder irrigation. Her urine cleared within a few days of irrigation, and the catheter was removed on day +72. Afterward, she was able to void clear urine but experienced vulvar itching and dysuria. The IV fluid infusion was discontinued, and she was advised to drink at least 3 liters of fluids daily. During a follow-up two days after catheter removal (day +74), she reported intermittent hematuria despite drinking plenty of fluids, with occasional passage of small blood clots. She subsequently received additional transfusions of irradiated red blood cells as needed, but her hematuria persisted.

**Figure 1.**
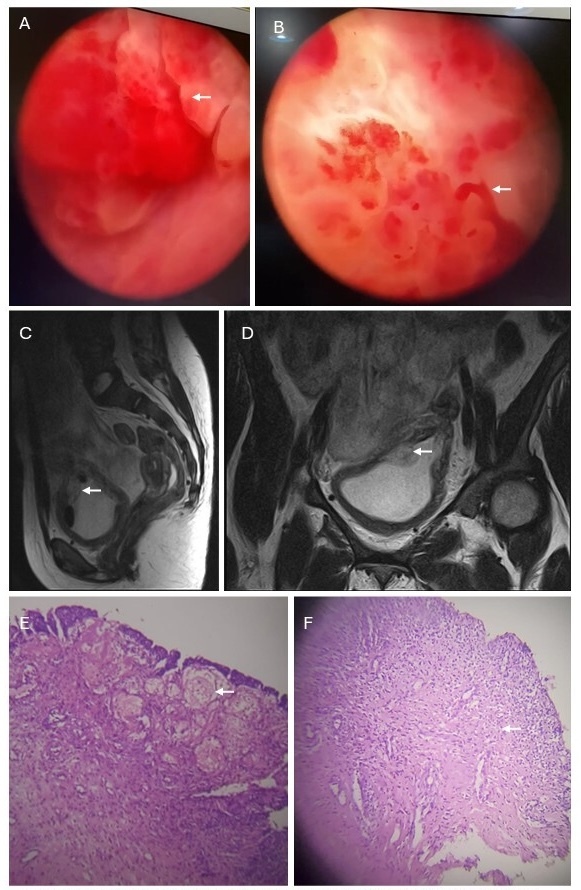
A) Coral-reef-like exophytic bladder mass with hypervascularized bladder wall. B) Bleeding point from bladder mass. C and D) Bladder mass with thickened bladder wall. E) Bladder tissue showing erosion of the urothelium with markedly congested blood vessels within the lamina propria (X40). F) Erosion with ulceration of the surface and oedema with inflammatory infiltrate in the lamina propria (X100)

On day +100, she eventually underwent a cystoscopy under conscious sedation, which revealed a hyper-vascularized bladder wall with two coral reef-like exophytic bladder masses on the right and left anterolateral wall with bleeding points (Figure 1A, 1B). The bleeding, friable masses were cauterized and thoroughly irrigated to achieve hemostasis, followed by complete bladder wash-out. Biopsy specimens of the masses were obtained for histological evaluation. A three-way silicone hematuric catheter was left in place for continuous bladder irrigation after the procedure. Further top-up transfusions with irradiated red cells were administered as needed. The catheter was removed after a week (day +106) when the urine cleared following discontinuation of bladder irrigation. Magnetic resonance imaging (MRI) of the abdomen, focusing on the bladder, showed diffuse bladder wall thickening with a focal mass (Figures 1C and 1D). The histology of the biopsy specimen from the bladder masses demonstrated dense stromal infiltration by inflammatory cells, mainly lymphocytes, suggesting a benign pseudotumor (Figures 1E and 1F).

Intermittent low-grade hematuria persisted afterward, but there was no decline in hemoglobin levels to justify further red cell transfusions. The platelet counts gradually increased without the need for transfusion, reaching 110,000/μL on day +105. She was regularly monitored with conservative management and liberal fluid intake until the hematuria resolved spontaneously by day +113 and has not recurred since.

## Discussion

Inflammatory pseudotumor of the urinary bladder is a rare benign condition of unknown cause. Predisposing factors include recurrent cystitis and previous urinary bladder surgery. Histologically, it is characterized by the presence of inflammatory cells with a variable fibrous response.^8,9^ Inflammatory pseudotumor related to HSCT is infrequently reported.^10^ Growing evidence indicates that SCD is also characterized by chronic inflammation and oxidative stress, which contribute to the development of chronic vasculopathy and various long-term complications.^11^ Possible complications of the proinflammatory state in SCD include calcifying fibrous pseudotumor (CFP), fibrous inflammatory lesions, fibromatosis, and other spindle cell neoplasms. Other pseudotumors, such as nodular fasciitis or inflammatory myofibroblastic tumor (IMT), have also been suggested.^12^ In our case, the pathology suggested a pseudotumor of the bladder. Understanding this condition and insisting on a definitive biopsy of mass lesions in the post-HSCT period can prevent unnecessary treatments such as radical surgery, chemotherapy, or radiotherapy.

Microscopic or macroscopic hematuria can occur in both adults and children with SCD and other medical conditions. Although hematuria is usually self-limiting, diagnostic evaluation is often necessary to identify its underlying cause and determine if treatment is needed.^13^ This usually involves a thorough assessment to narrow down the broad differential diagnoses, including a detailed clinical history followed by relevant laboratory tests with or without imaging studies. Hematuria after allogeneic HSCT for SCD may result from the underlying disease or as a complication of the transplant process.^3^ Common renal complications in SCD before transplant include renal papillary necrosis, sickle cell nephropathy, kidney stones, urinary tract infections caused by bacteria, viruses, or parasites, and mass lesions in the kidney or urinary system tract.^4^ Multiple causes can coexist, and a high level of suspicion is often necessary to identify rare causes. Renal papillary necrosis affects 15-30% of individuals with SCD, mainly in children.^5^ It results from the obstruction of blood vessels in the kidney, leading to infarction and bleeding. The typical clinical presentation - sudden-onset hematuria - is painless , but can also be associated with flank pain. The presence of a fever indicates a possible superimposed infection, which can worsen the hematuria or cause other complications of SCD, such as a bone pain crisis. Another common cause of hematuria in this population is sickle cell nephropathy, a significant cause of morbidity and mortality due to chronic kidney disease damage.^3^ Proteinuria is the most common sign of sickle cell nephropathy, but it can also be associated with hematuria. It is characterized by changes in the kidney’s structure and function, including hyperfiltration, vasculopathy, and impaired urine concentration regulation.^4^ Renal stones are also a frequent cause of hematuria, which can occur anywhere along the urinary tract, including the renal pelvis, ureter, and bladder. They are common in patients with SCD, caused by excess uric acid or calcium oxalate in the urine.^3,4^ Hematuria may cause pain depending on where the stone forms or moves, and can lead to repeated infections. SCD is associated with significant immune deficiency, which increases the risk of severe infections post-transplant.^14^

The post-transplant period is a highly immunosuppressive state. Urinary tract infections (UTIs) caused by various pathogens can present with hematuria. Usually, the latter is accompanied by irritative urinary symptoms, including increased frequency, dysuria, and urgency. The urinary bladder is often involved in infection, leading to cystitis. Multiple bacterial organisms have been linked to UTIs and are primarily diagnosed in females. Other common infectious causes of hematuria include viral cystitis caused by BK virus and adenovirus, which are frequently observed in SCD patients with suppressed immunity due to immunosuppressive therapy post-HSCT.^3^ Infectious causes of hematuria can overlay other causes, such as renal papillary necrosis and kidney stones, further intensifying hematuria. In tropical areas, parasitic infections like urinary schistosomiasis caused by Schistosoma haematobium are common, and usually show symptoms at the end of urination, such as hematuria.^15^

Cyclophosphamide is typically administered at high doses in most myeloablative conditioning regimens for HLA-matched allogeneic HSCT in SCD. It is also prescribed post-transplant in haploidentical transplant recipients to prevent GVHD.^16^ The use of cyclophosphamide in HSCT has been associated with a high risk of hemorrhagic cystitis, which can be prevented with hyperhydration and mesna. However, hematuria may still occur despite these preventative measures, usually within a few days to weeks after the infusion. Hemorrhagic cystitis can cause obstructive kidney injury when blood clots in the bladder block the outflow tract. The causes of hemorrhagic cystitis are usually multifactorial, often linked to cyclophosphamide use or the reactivation of viral infections such as BK virus, adenovirus, and cytomegalovirus. Treatments include hyperhydration, diuresis, and bladder irrigation using a three-way bladder catheter.^7^

Although infrequent, both benign and malignant mass lesions should be included in the differential diagnoses of hematuria. Renal medullary carcinoma has been linked to SCD and is commonly observed in children.^4^ It usually presents with hematuria and abdominal pain. It is a rapidly progressing condition that requires prompt identification and aggressive symptom management.^17^ Other renal malignancies reported in SCD include transitional cell carcinoma and renal clear cell carcinoma.^4^ Secondary malignancies have been observed as a late effect of cancer immunotherapy.^18^ Despite the broad differential diagnosis of hematuria in patients with SCD undergoing HSCT, it is crucial to follow a systematic evaluation approach that considers common causes but also remains alert for rare etiologies. The assessment of a patient with SCD presenting with hematuria should start with a detailed clinical history and physical examination. Initial tests should include a complete blood count, coagulation screening, and renal function tests to detect bleeding disorders and renal issues, respectively. A renal ultrasound can identify renal papillary necrosis, stones, or masses. However, additional imaging methods such as computed tomography (CT) or magnetic resonance (MR) urography may be necessary, as these provide higher detection rates. Cystoscopy can be performed after a urological assessment, following review of the initial diagnostic tests. In summary, this report emphasizes that hematuria after HSCT for SCD requires proper evaluation to ensure appropriate treatment and to avoid unnecessary procedures for this concerning symptom.

### Author contributions per CRediT

Writing – original draft: Yusuf Adelabu (Equal), Adetola A. Kassim (Equal). Writing – review & editing: Ugonna O. Fakile (Equal), Wale Q. Oladimeji (Equal), Kayode Oseni (Equal), Moses A. Ogunjimi (Equal), Titilayo G. Bamigboye (Equal), Blessing N. Aziken-John (Equal), Amina Enegbuma (Equal), Zainab Olayiwola (Equal), Olufunto Kalejaiye (Equal), Titilope A. Adeyemo (Equal), Ann Ogbenna (Equal), Karina Wilkerson (Equal), Edamisan Temiye (Equal), Adeseye M. Akinsete (Equal).

### Disclosure statement

The authors report no conflicts of interest. The authors alone are responsible for the content and writing of this article.

### AI

Generative AI and AI-Assisted Technologies were not used in the preparation of this manuscript.

## Data Availability

Data sharing is not applicable to this article.
